# BMP10 Knockdown Modulates Endothelial Cell Immunoreactivity by Inhibiting the HIF‐1α Pathway in the Sepsis‐Induced Myocardial Injury

**DOI:** 10.1111/jcmm.70232

**Published:** 2024-11-29

**Authors:** Huan Guan, Jingyun Fang

**Affiliations:** ^1^ Department of Emergency Ganzhou People's Hospital Ganzhou Jiangxi China

**Keywords:** BMP10, HIF‐1α pathway, sepsis‐induced myocardial injury

## Abstract

Sepsis is a life‐threatening syndrome triggered by a cascade of dysregulated immune responses. Sepsis‐induced myocardial injury (SIMI) substantially impacts the survival time of septic patients. However, the molecular mechanisms underlying the pathology of SIMI remain unclear. Immune‐related differentially expressed genes in SIMI were identified through RNA sequencing and bioinformatics analysis. The expression levels of hub genes were detected using reverse transcription quantitative PCR. BMP10 was knocked down in the lipopolysaccharide‐induced mouse and cardiac microvascular endothelial cell (CMEC) models, and its functions were assessed by a series of in vitro and in vivo assays. Cell adhesion and HIF‐1 pathway‐associated protein expressions were measured by western blot. Fenbendazole‐d3 was used to investigate whether BMP10 influenced SIMI development by modulating the HIF‐1 pathway. Six key genes were screened, of which BMP10, HAMP, TRIM5, and MLANA were highly expressed, and PTPRN2 and AVP were lowly expressed. BMP10 knockdown ameliorated histopathological changes and inhibited apoptosis and CMEC immune infiltration in SIMI. BMP10 knockdown reduced inflammatory factor (IL‐6, MCP‐1, IFN‐β, and CCL11) levels and protein expressions of cell adhesion‐related molecules (VCAM‐1 and ICAM‐1). Mechanistically, the HIF‐1 pathway agonist, Fenbendazole‐d3, significantly reversed the inhibitory effects of BMP10 knockdown on SIMI in vitro, indicating that BMP10 knockdown impeded the development of SIMI by suppressing the HIF‐1α pathway. BMP10 knockdown blocks SIMI progression by inhibiting the HIF‐1α pathway, which provides a new potential therapeutic strategy for SIMI treatment.

## 1 Introduction

Sepsis, a clinical syndrome marked by a dysregulated host response to infection, is a major contributor to mortality worldwide [1]. Pathophysiology of septic multiorgan failure involves complex interactions between pathogens and the host immune system. The immune response triggers severe macro‐ and microcirculatory dysfunction, resulting in profound global hypoperfusion and injury to multiple organs [2, 3]. Heart dysfunction, particularly sepsis‐induced myocardial injury (SIMI), is recognised as a crucial factor contributing to septic mortality [4]. About 40%–50% of sepsis patients experience SIMI, presenting with symptoms such as arrhythmia, hypotension, and heart failure [5]. Dysregulated immune response leading to excessive inflammation is a major contributor to SIMI [6]; however, the exact molecular mechanisms are still unclear.

Bone morphogenetic proteins (BMPs), the multifunctional cytokines, are crucial for the development and differentiation of various tissues [7]. Currently, approximately 20 BMPs have been identified, constituting the largest subgroup in the TGF‐β family [8]. BMP10 is a cardiac peptide growth factor that is abundant in the heart and is pivotal to vascular remodelling and cardiac development [9]. BMP10 initiates its intracellular signalling by binding to a receptor complex comprising activin receptor‐like kinase 1 with either morphogenetic protein receptor type II or activin receptor type 2A [10]. Earlier investigations have illustrated that mice overexpressing BMP10 develop cardiac hypertrophy characterised by excessive ventricular trabeculae, while mice deficient in BMP10 exhibit hypoplastic and thin ventricles with minimal ventricular trabeculae [11, 12]. Additionally, BMP10 is identified as a new gene for dilated cardiomyopathy [13]. By activating the signal transducer and activator of the transcription 3 signalling pathway, BMP10 mitigates doxorubicin‐induced cardiac injury [14]. However, there are limited studies on the functions of BMP10 in SIMI. Furthermore, BMP10 is involved in the immune process. BMP10 may regulate the role of ATF3 in immune response through the Smad‐dependent pathway [15]. Through a Smad4‐dependent pathway, BMP9/BMP10/ALK1 signalling regulate the particular gene expression programme and survival of resident macrophage Kupffer cells [16]. Therefore, it is critical to investigate the roles and molecular mechanisms of BMP10, an immune‐related gene, in SIMI.

Hypoxia‐inducible factor (HIF), a heterodimeric transcription factor, is a member of the bHLH‐PAS (basic‐helix–loop–helix‐Per‐Arnt‐Sim) family. It is essential for orchestrating the adaptive response to hypoxia [17]. HIF comprises an oxygen‐sensitive HIF‐α subunit and a HIF‐β subunit [18]. Study has revealed that decreased HIF‐1α expression after ischemia–reperfusion injury may exacerbate myocardial injury and vascular dysfunction [19]. HIF‐1α‐deficient myeloid cells have pro‐inflammatory properties [20]. In addition, during sepsis, the restriction of glycolysis led to neutrophil immunosuppression and may be regulated by LDHA downregulation mediated by the PI3K/Akt‐HIF‐1α pathway [21]. However, whether BMP10 influences SIMI progression by regulating the HIF‐1α pathway in SIMI is unclear.

In this investigation, SIMI‐associated differentially expressed genes (DEGs) in the lipopolysaccharide (LPS)‐induced SIMI mouse model were identified by RNA sequencing. DEGs and immune infiltration‐related genes were intersected and analysed using bioinformatics. Ultimately, six key genes were identified. BMP10 expression was elevated in SIMI and knockdown of BMP10 inhibited apoptosis, inflammation, and cell adhesion processes in LPS‐induced cardiac microvascular endothelial cells (CMECs). These results were also validated in vivo. Further investigations demonstrated that knockdown of BMP10 inhibited SIMI development by suppressing the HIF‐1α pathway. Our study offers novel viewpoints into the pathogenesis and treatment of SIMI.

## 2 Materials and Methods

### 2.1 Construction of SIMI Mouse Model

All animal experiments adhered to the National Institutes of Health Guidelines for the Care and Use of Laboratory Animals. A total of 24 male C57BL/6 mice (SPF Biotechnology Co. Beijing, China; aged 6–8 weeks, 18–20 g) were housed in an aseptic environment with a 12‐h light/dark cycle, temperature of 22°C ± 1°C, and adequate food and water. After 7 days of acclimatisation feeding, construction of the SIMI mouse model was initiated as previously described [22]. The SIMI model was induced by an abdominal injection of 10 mg/kg LPS (Sigma‐Aldrich, St. Louis, MO, USA). The control group mice received an equivalent volume of saline injection. Mice were randomly separated into four groups, including control, LPS, LPS + AAV‐NC and LPS + AAV‐BMP10 groups. Furthermore, 48 h before LPS treatment, mice in LPS + AAV‐NC and LPS + AAV‐BMP10 groups were intraperitoneally injected with 5 μL of AAV‐NC or AAV‐ BMP10 (6.25 × 10^12^ viral genomes/mL). The mice were killed under inhalation anaesthesia with isoflurane (2%, Woruide, Shenzhen, China) and then serum and cardiac tissue samples were collected for subsequent experiments.

### 2.2 RNA Sequencing

The total RNA was extracted from the myocardial tissues utilising the TRIzol reagent (Thermo Fisher Scientific, MA, USA). The quality of RNA was evaluated using a 2100 Bioanalyzer (Agilent Technologies, Santa Clara, CA, USA). Then, the mRNA underwent enrichment, fragmentation and synthesis into double‐stranded cDNA. The cDNA library was constructed and sequenced by the Shanghai Majorbio Bio‐pharm Biotechnology Co. Ltd. (Shanghai, China). Genes meeting the criteria of |log2FoldChange| > 1 and *p* < 0.05 were regarded as significant DEGs for subsequent analysis. The DEGs identified in SIMI were then intersected with the genes associated with immune infiltration in the GeneCards database (https://www.genecards.org/) to obtain the immune infiltration‐related DEGs in SIMI. Using the ggplot2 and pheatmap packages in R software (version 4.2.3), volcano plot and heatmap of DEGs were developed. In the DAVID database (https://david.ncifcrf.gov), DEGs were subjected to functional enrichment analysis for the Gene Ontology (GO) and Kyoto Encyclopedia of Genes and Genomes (KEGG).

### 2.3 Isolation of CMECs

Mice were anaesthetised, and their hearts were swiftly excised. The left ventricle was treated with 70% ethanol for 30 s, followed by extensive washing with calcium‐free Krebs–Henseleit bicarbonate buffer. Segments of the left ventricle were subjected to incubation with 0.2% collagenase type I (Sigma‐Aldrich) for 10 min, followed by treatment with 0.25% trypsin (Hyclone, Logan, UT, USA) for 5 min at 37°C in a shaking bath. Dissociated cells were filtered and further cultivated in Dulbecco's modified Eagle's medium (DMEM; Thermo Fisher Scientific, Waltham, MA, USA) supplemented with microvascular growth supplements (Thermo Fisher Scientific), 5% foetal bovine serum (FBS), and 1% penicillin–streptomycin. The cells were grown under 5% CO_2_ at 37°C and cells from the third generation were chosen for the experiment.

### 2.4 Cell Transfection

Three types of small interfering RNA (siRNA) targeting BMP10 (si‐BMP10‐1, si‐BMP10‐2 and si‐BMP10‐3) and a negative control (si‐NC) were synthesised by RiboBio (Guangzhou, China). CMECs were transfected with 10 nM of si‐BMP10‐1, si‐BMP10‐2, si‐BMP10‐3 or si‐NC utilising Lipofectamine 3000 (Thermo Fisher Scientific) following the manufacturer's recommendations. After 24 h of incubation, transfected cells were exposed to 10 μg/mL LPS or LPS + 1 μM Fenbendazole‐d3 (HIF‐1 pathway agonist) for 24 h. Cells in the control group were incubated in medium with equal amounts of sterile deionised water.

### 2.5 Reverse Transcription Quantitative Real‐Time PCR (RT‐qPCR)

Total RNA was isolated using TRIzol reagent (Thermo Fisher Scientific). Subsequently, after converting RNA into cDNA, RT‐qPCR was carried out using SYBR Green Mix (Takara, Dalian, China) on a 7500 real‐time PCR system (Thermo Fisher Scientific). The 2^−ΔΔCt^ method was used to quantify relative mRNA expression, with GAPDH as a housekeeping gene. Primer sequences are provided in Table S1.

### 2.6 Cell Counting Kit‐8 (CCK‐8) Assay

The cell viability was evaluated by employing the CCK‐8 kit (Solarbio, Beijing, China). In a 96‐well plate, 1 × 10^5^ cells were planted into each well. The 10 μL of CCK‐8 solution was added to each well and incubated for 2 h after the initial 24‐h incubation. Using a microplate reader (DALB, Shanghai, China), absorbance at 450 nm was measured.

### 2.7 Flow Cytometry

Following 3‐min centrifugation at 1000 ***g*** at room temperature, the cells were resuspended in 1× Annexin V Binding Buffer. Then, 5 μL of Annexin V‐FITC with 5 μL of propidium iodide solution (Beyotime, Shanghai, China) was introduced into the mixture, and the cells were incubated in the dark for 30 min. A FACScan flow cytometer (Becton, Dickinson and Company, NJ, USA) was used to measure apoptosis.

### 2.8 Enzyme‐Linked Immunosorbent Assay (ELISA)

The concentrations of interleukin 6 (IL‐6), monocyte chemoattractant protein‐1 (MCP‐1), IFN‐beta (IFN‐β), chemokine eotaxin‐1 (CCL11), creatine kinase‐MB (CK‐MB), lactate dehydrogenase (LDH) and cardiac troponin T (cTnT) were measured in the mouse serum or cell culture medium employing the ELISA kits (Esebio Biotechnology Co. Ltd. Shanghai, China) based on the manufacturer's instructions.

### 2.9 Western Blot Assay

The cardiac tissues and cells were collected and then lysed using RIPA lysis buffer (Solarbio) to isolate total protein. Proteins were separated on 10% SDS‐polyacrylamide gel electrophoresis (SDS‐PAGE) gels and subsequently transferred to polyvinylidene difluoride membranes (Roche, Basel, Switzerland) using electro‐transfer. After blocking with 5% nonfat milk at room temperature, the membranes were treated with diluted primary antibodies, including VCAM‐1 (1:2000 dilution; Abcam, Cambridge, UK), ICAM‐1 (1:2000 dilution; Abcam), HIF‐1α (1:2000 dilution; Abcam) and GAPDH (1:2000 dilution; Abcam). Afterwards, the membranes were exposed to an HRP‐conjugated secondary antibody for 1 h. Protein bands were visualised using enhanced chemiluminescence reagents (Amersham, Little Chalfont, UK) and the levels of protein expression were quantified using ImageJ software.

### 2.10 Hematoxylin and Eosin (HE) Staining

Myocardial tissues of mice were fixed with 4% paraformaldehyde and then were washed, dehydrated, cleaned, soaked in wax, embedded and sliced into 4 μm sections. Then, paraffin sections were stained with haematoxylin and eosin following the manufacturer's instructions. Five different fields of view were photographed with a BX‐51 light microscope (Olympus, Tokyo, Japan) to observe pathological changes in cardiac tissues.

### 2.11 Terminal Deoxynucleotidyl‐Transferase‐Mediated dUTP Nick End Labelling (TUNEL) Staining

The apoptosis ratio of the cardiomyocytes was measured using a TUNEL assay kit (Roche Diagnostics, Basel, Switzerland). To identify apoptotic cell nuclei, TUNEL was carried out using fluorescein‐dUTP for 1 h at 37°C. All cell nuclei were then stained for 5 min at room temperature using DAPI. TUNEL‐positive cells were analysed using a microscope (Olympus). TUNEL‐positive cells appeared red. The apoptosis rate was calculated using the formula: TUNEL‐positive nuclei/DAPI‐stained nuclei × 100%.

### 2.12 Immunohistochemical (IHC) Staining

The paraffin‐embedded mouse cardiac tissues were sectioned into 4 μm slices and then rehydrated through xylene deparaffinisation followed by gradient ethanol immersion. A citrate antigen retrieval solution was used for antigen retrieval, and 3% hydrogen peroxide was used to block endogenous peroxidase. The sections underwent blocking with goat serum for 1 h, followed by overnight incubation at 4°C with macrophage primary antibody (dilution 1:500; Abcam). Afterwards, the sections were treated with an HRP‐labelled secondary antibody for 30 min and counterstained with haematoxylin for 10 min. The tissues were observed under a light microscope (Olympus).

### 2.13 Statistical Analysis

GraphPad Prism 7.0 was used for statistical analysis. Results are presented as mean ± standard deviation (SD). Statistical significance between two groups was assessed using the *t*‐test, while multiple group comparisons were conducted using Analysis of Variance (ANOVA) followed by Tukey's multiple comparison test. The *p* < 0.05 was considered statistically significant.

## 3 Results

### 3.1 DEGs Were Screened by RNA Sequencing

**FIGURE 1:**
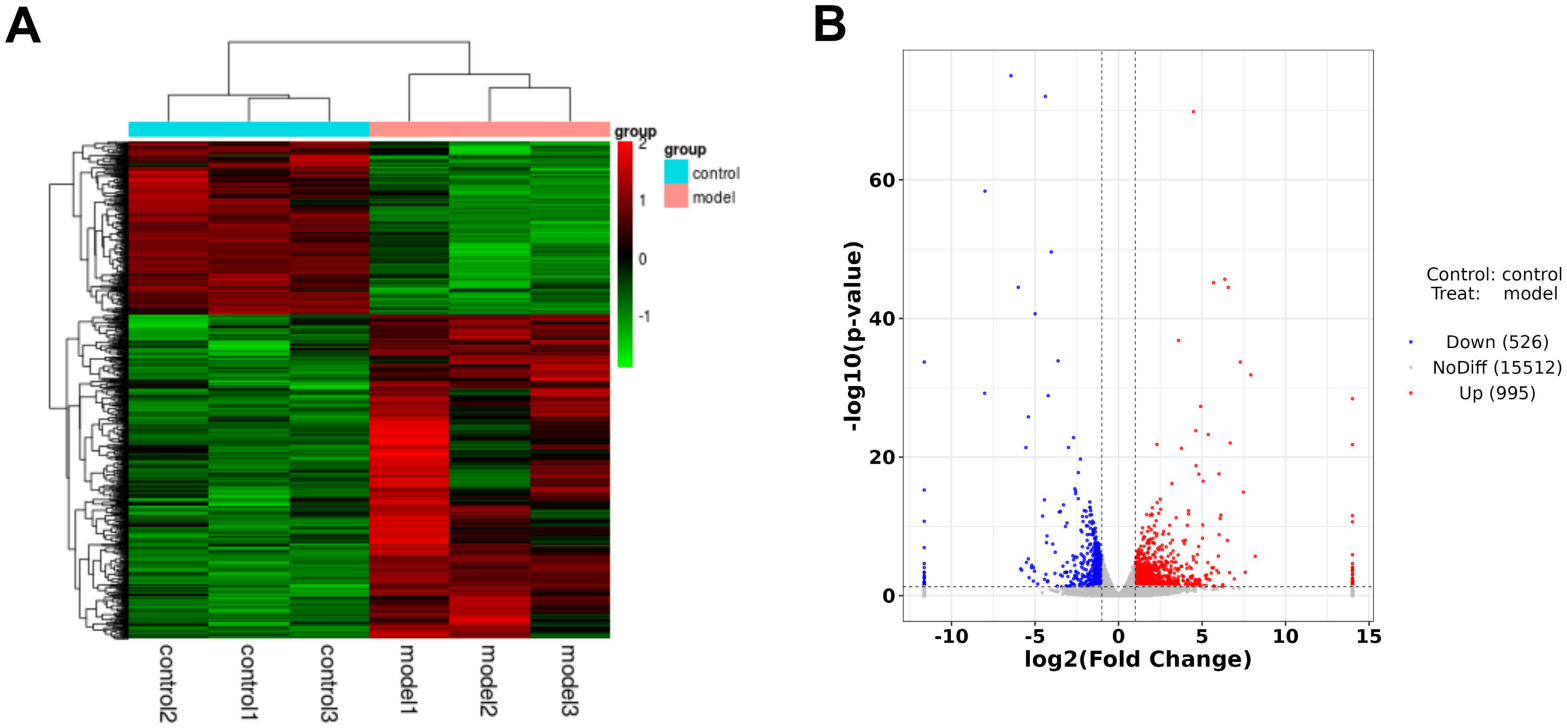
DEGs were identified through RNA sequencing. (A) Bidirectional clustering analysis of concatenated samples of differential genes from control and SIMI model groups was performed using the Pheatmap package in R language. (B) The R ggplot2 package was utilised to generate volcano plot of DEGs. DEGs, differentially expressed genes; SIMI, sepsis‐induced myocardial injury.

Tissues from the hearts of normal control and SIMI model mice were subjected to RNA sequencing. Bioinformatics analysis uncovered notable alterations in the entire transcriptome between the control and myocardial injury groups, with cluster analysis yielding distinctly categorised and well‐parallelised results (Figure 1A). Based on |log_2_FC| > 1 and *p* < 0.05, there were 1521 DEGs, comprising 995 upregulated and 526 downregulated genes (Figure 1B). Table S2 demonstrated the top 10 up‐ and down‐regulated DEGs.

### 3.2 Functional Enrichment Analysis of DEGs

**FIGURE 2:**
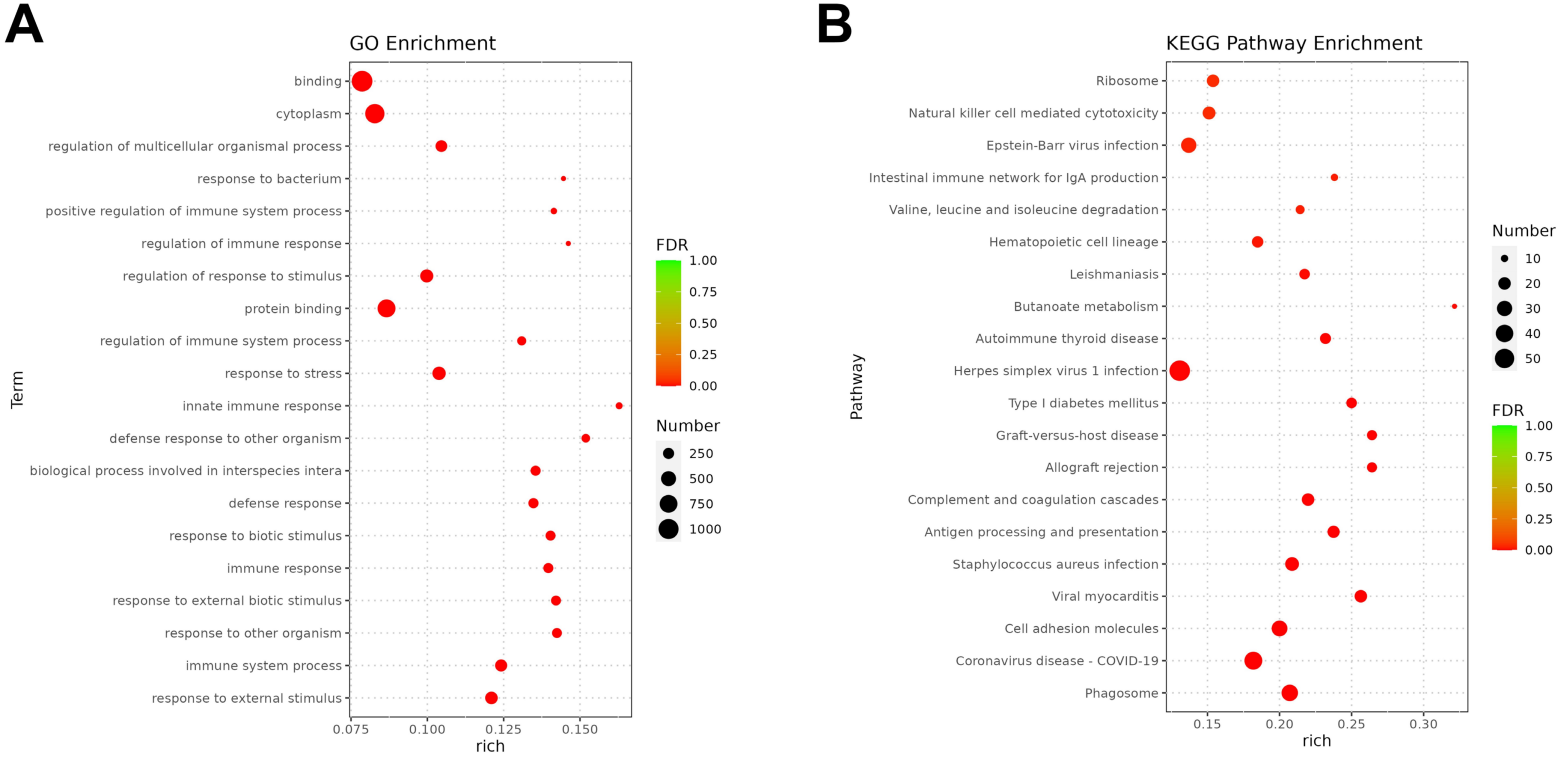
Functional enrichment analysis of DEGs. (A) A bubble plot for GO annotation analysis. (B) A bubble plot for KEGG function enrichment analysis. DEGs, differentially expressed genes; GO, gene ontology; KEGG, Kyoto Encyclopedia of Genes and Genomes.

The potential biological functions of the DEGs were further explored. The results indicated that the DEGs were mostly correlated with innate immune response, defence response to other organism, immune system process and protein binding in the GO classification (Figure 2A). The results from the KEGG analysis illustrated that DEGs were primarily involved in the butanoate metabolism, viral myocarditis, cell adhesion molecules and Herpes simplex virus 1 infection (Figure 2B).

### 3.3 RT‐qPCR Detection of Key Gene Expression Levels

**FIGURE 3:**
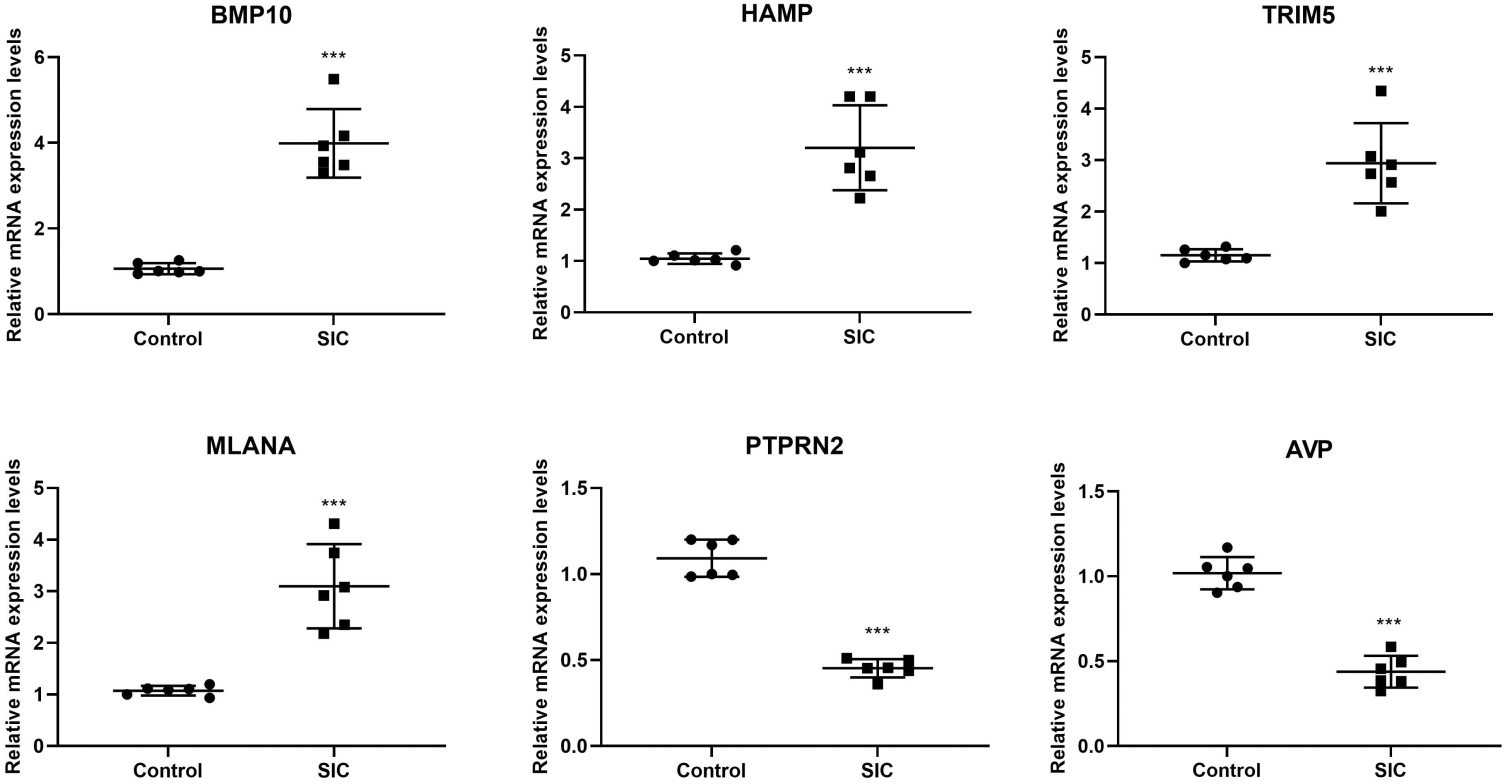
RT‐qPCR was used to detect the expression levels of key genes, including BMP10, HAMP, TRIM5, MLANA, PTPRN2 and AVP. ***p < 0.001 versus Control group. RT‐qPCR, reverse transcription quantitative PCR.

The DEGs were intersected with the immune infiltration‐related genes, resulting in a total of 505 genes (Figure S1). From this intersection, BMP10, HAMP, TRIM5, MLANA, PTPRN2 and AVP were identified as key genes based on their significant differences in expression levels. Then, the mRNA levels of these key genes were confirmed using RT‐qPCR in CMECs. The findings demonstrated that BMP10, hepcidin antimicrobial peptide (HAMP), tripartite motif containing 5 (TRIM5) and melan‐A (MLANA) expressions were markedly elevated, while the protein tyrosine phosphatase, receptor type N2 (PTPRN2) and arginine vasopressin (AVP) were substantially reduced in the sepsis‐induced cardiomyopathy (SIC) group in comparison with the control group (Figure 3).

### 3.4 Knockdown of BMP10 Suppressed Apoptosis, Inflammation and Cell Adhesion in CMECs

**FIGURE 4:**
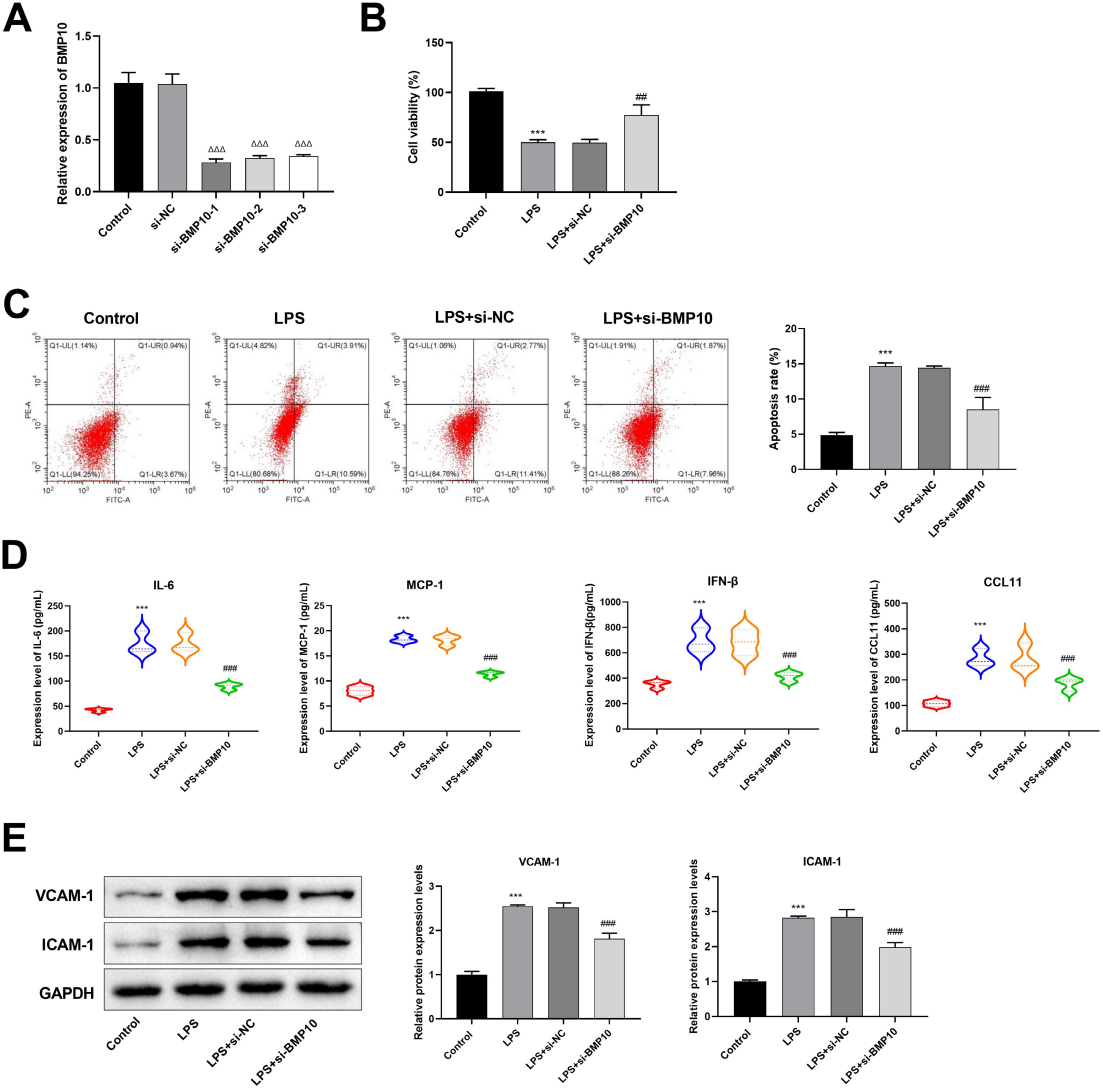
BMP10 knockdown inhibited apoptosis, inflammation and cell adhesion in CMECs. (A) After the CMECs were transfected with si‐BMP10‐1, si‐BMP10‐2 and si‐BMP10‐3, RT‐qPCR was used to assess the knockdown efficiency of BMP10. ^ΔΔΔ^ *p* < 0.001 versus si‐NC group. (B) Cell viability in the control, LPS, LPS + si‐NC and LPS + si‐ BMP10 groups was assessed by CCK‐8 assay. (C) Apoptosis was measured using flow cytometry in the four groups. (D) Levels of inflammatory factors, including IL‐6, MCP‐1, IFN‐β and CCL11 were detected by ELISA assay. (E) Protein levels of cell adhesion molecules, including VCAM‐1 and ICAM‐1were evaluated by western blot analysis. ***p < 0.001 versus control group; ^##^ *p* < 0.01 hematoxylin and eosin, ^###^ *p* < 0.001 versus LPS + si‐NC group. CMECs, cardiac microvascular endothelial cells; CCK‐8, cell counting kit‐8; ELISA assay, enzyme‐linked immunosorbent assay; RT‐qPCR, reverse transcription quantitative PCR.

In CMECs, we knocked down BMP10 to assess its role in SIMI. RT‐qPCR revealed that the BMP10 expression was notably decreased in the LPS + si‐BMP10‐1, LPS + si‐BMP10‐2 and LPS + si‐BMP10‐3 groups in contrast to that in the LPS + si‐NC group. Subsequent experiments were conducted using si‐BMP10‐1 due to its superior transfection efficiency compared to other variants of si‐BMP10 (Figure 4A). CCK‐8 revealed that the cell viability in the LPS group was considerably lower than that of the control group. Whereas, by comparison to that the LPS + si‐NC group, cell viability was considerably increased in the LPS + si‐BMP10 group (Figure 4B). The results from the flow cytometry illustrated that LPS treatment induced apoptosis, while knockdown of BMP10 significantly inhibited apoptosis (Figure 4C). ELISA was employed to measure the levels of the inflammatory factors, including IL‐6, MCP‐1, IFN‐β and CCL11. The results indicated that the IL‐6, MCP‐1, IFN‐β and CCL11 levels were substantially elevated in the LPS group as opposed to that in the control group. Knockdown of BMP10 inhibited these inflammatory factor levels (Figure 4D). Additionally, western blot showed that the protein expressions of cell adhesion molecules, including VCAM‐1 and ICAM‐1 were substantially increased in the LPS group in contrast to that in the control group, and this trend was greatly overturned by the BMP10 knockdown (Figure 4E).

### 3.5 Knockdown of BMP10 Inhibited Apoptosis, Inflammation and Cell Adhesion in the SIMI Mouse Model

**FIGURE 5:**
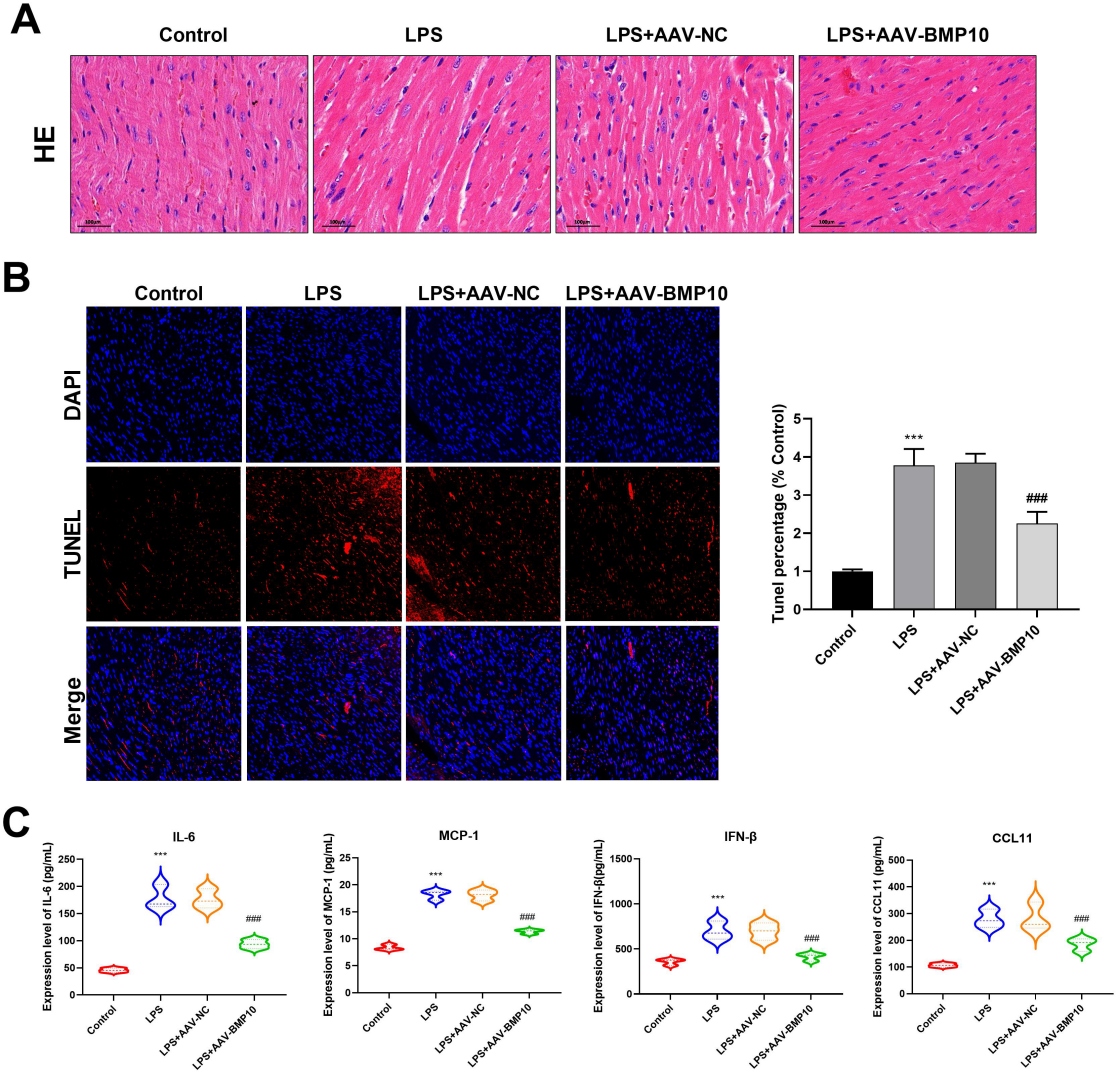
Knockdown of BMP10 ameliorated pathological changes and inhibited apoptosis and inflammatory responses in SIMI mice model (*n* = 6 mice per group). (A) Histopathological changes in the cardiac tissues of mice were examined using HE staining in the control, LPS, LPS + AAV‐NC and LPS + AAV‐BMP10 groups. Magnification: 400×, Scale: 100 μm. (B) Fluorescent TUNEL labelling (red) and DAPI nuclear staining (blue) were employed to respectively detect apoptotic cells and visualise cell nuclei. (C) The ELISA assay was employed to measure the levels of inflammatory factors (IL‐6, MCP‐1, IFN‐β and CCL11) in the SIMI mice model. ***p < 0.001 versus control group: ^###^ *p* < 0.001 versus LPS + AAV‐NC group. ELISA, enzyme‐linked immunosorbent assay; HE staining, hematoxylin and eosin staining; SIMI, sepsis‐induced myocardial injury; TUNEL, terminal deoxynucleotidyl‐transferase‐mediated dUTP nick end labelling.

**FIGURE 6:**
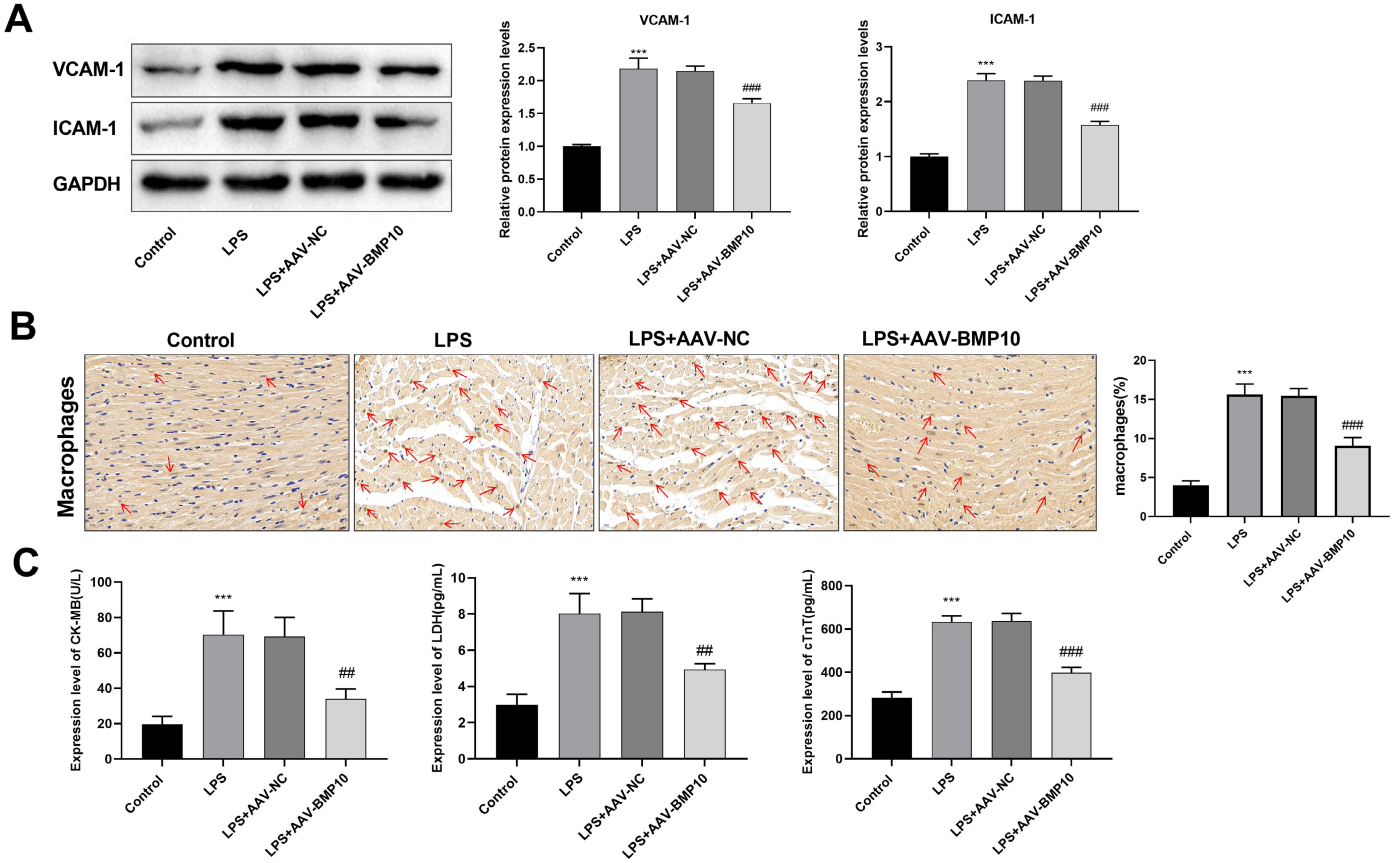
Knockdown of BMP10 inhibited cell adhesion and immune cell infiltration in SIMI mice model. (A) Western blot was used to examine the expression levels of cell adhesion molecules VCAM‐1 and ICAM‐1. (B) IHC staining analysis of peripheral immune cell infiltration in mouse heart. Magnification: 400×, Scale bar: 20 μm. Red arrows indicate macrophages. (C) ELISA assay was performed to quantify the expression levels of myocardial injury markers (CK‐MB, LDH and cTnT) in the mouse heart. ***p < 0.001 versus control group; ^##^ *p* < 0.01, ^###^ *p* < 0.001 versus LPS + AAV‐NC group. ELISA, enzyme‐linked immunosorbent assay; IHC staining, immunohistochemical staining; SIMI, sepsis‐induced myocardial injury.

The function of BMP10 in SIMI was further explored in vivo. The mice in the LPS group displayed aggregated inflammatory cells in the heart tissues, disordered arrangement of myocardial fibres and mild congestion. Compared to the LPS + AAV‐NC group, mice subjected to BMP10 knockdown displayed reduced tissue inflammatory cells and well‐arranged cardiac muscle fibres (Figure 5A). TUNEL staining revealed a notable elevation in the apoptosis rate in the LPS group relative to that in the control group. Conversely, the apoptosis rate was notably decreased in the LPS + AAV‐BMP10 group in comparison with that in the LPS + AAV‐NC group (Figure 5B). ELISA indicated that the IL‐6, MCP‐1, IFN‐β and CCL11 levels were substantially higher in the LPS group than that in the control group. Knockdown of BMP10 decreased these inflammatory factor levels (Figure 5C). The protein expressions of VCAM‐1 and ICAM‐1 were significantly elevated after treatment with LPS. However, BMP10 knockdown led to a reduction in the VCAM‐1 and ICAM‐1 protein expressions (Figure 6A). IHC staining was used to analyse peripheral immune cell infiltration in the mouse heart. The results showed a noteworthy increase in the accumulation of myocardial neutrophils and macrophages in mice in the LPS group relative to that in the control group, and this was notably inverted by BMP10 knockdown (Figure 6B). Moreover, the levels of myocardial injury markers in mouse hearts were detected by ELISA. The findings indicated that the CK‐MB, LDH and cTnT expressions were greatly elevated in the LPS group corresponding to that the control group. Conversely, these marker levels were obviously reduced in the LPS + AAV‐BMP10 group in relation to that the LPS + AAV‐NC group (Figure 6C).

### 3.6 Knockdown of BMP10 Blocked SIMI Progression by Inhibiting the HIF‐1α Pathway

**FIGURE 7:**
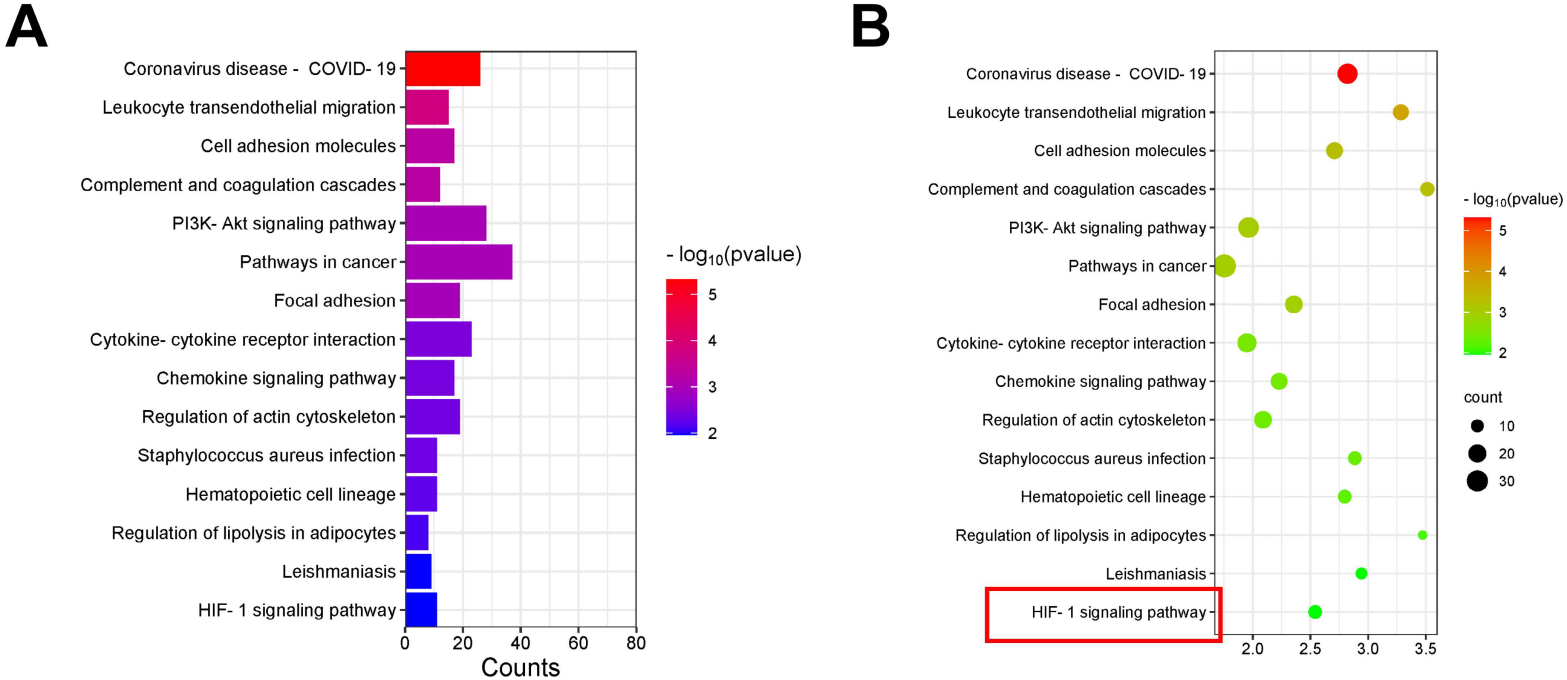
KEGG functional enrichment analysis for BMP10‐related immune infiltration DEGs in SIMI. (A) Histogram of KEGG functional enrichment analysis. (B) Bubble plot of KEGG functional enrichment analysis. KEGG, Kyoto Encyclopedia of Genes and Genomes; SIMI, sepsis‐induced myocardial injury.

**FIGURE 8:**
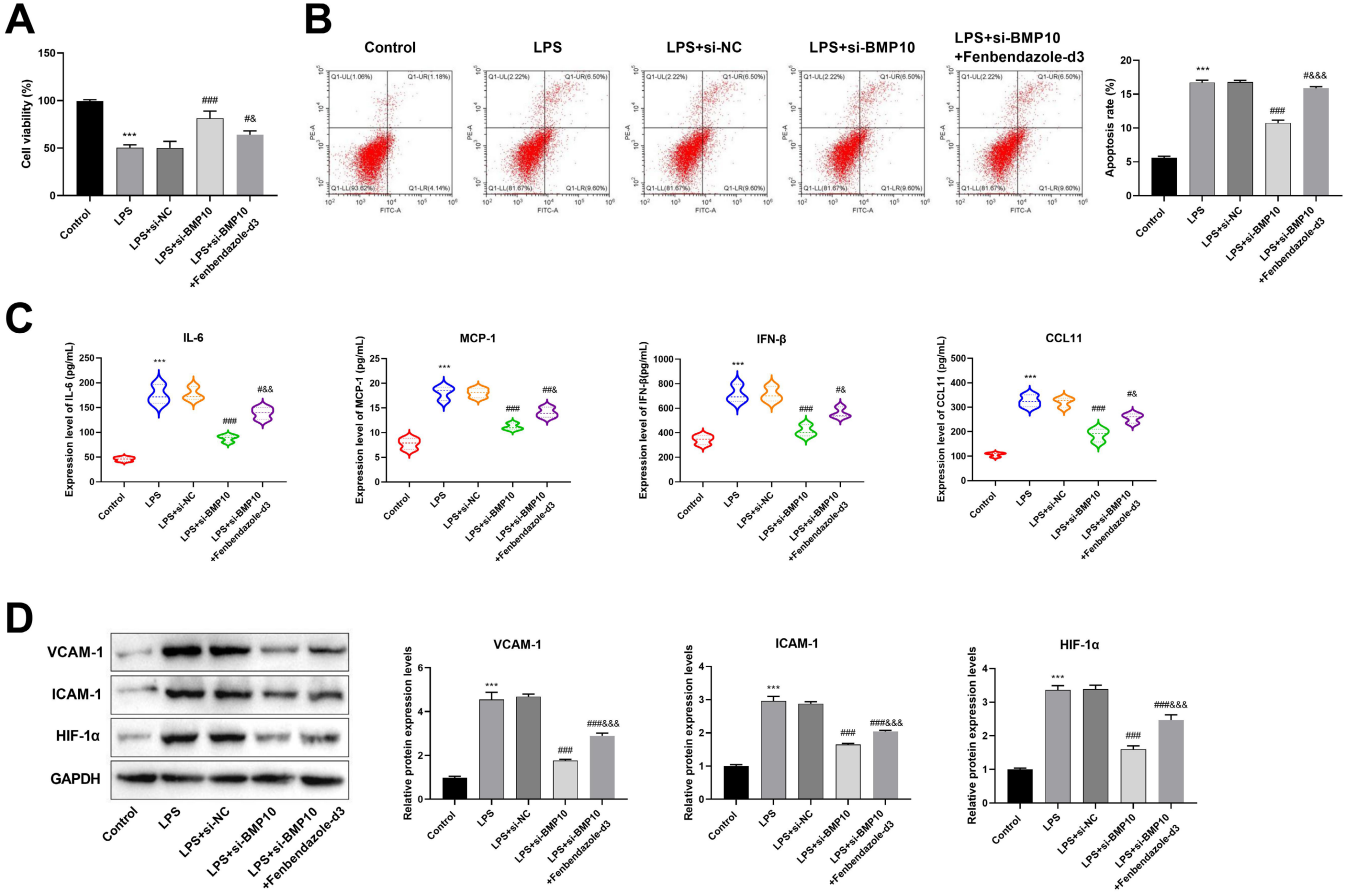
Knockdown of BMP10 suppressed SIMI development by suppressing the HIF‐1α pathway. (A) Cell viability in the control, LPS, LPS + si‐NC, LPS + si‐BMP10 and LPS + si‐BMP10 + Fenbendazole‐d3 groups was measured by CCK‐8 assay. (B) Flow cytometry was performed to assess apoptosis in five groups. (C) The levels of inflammatory factors (IL‐6, MCP‐1, IFN‐β and CCL11) were measured using the ELISA assay. (D) The expression levels of cell adhesion molecules VCAM‐1, ICAM‐1 and key proteins of the HIF‐1 pathway were assessed using western blot analysis. ***p < 0.001 versus control group; ^#^ *p*＜0.05, ^##^ *p*＜0.01, ^###^ *p* < 0.001 versus LPS + si‐NC group; ^&^ *p* < 0.05, ^&&^ *p* < 0.01 and ^&&&^ *p* < 0.001 versus LPS + si‐BMP10 group. CCK‐8, cell counting kit‐8; ELISA, enzyme‐linked immunosorbent assay; SIMI, sepsis‐induced myocardial injury.

The molecular mechanism by which BMP10 knockdown inhibited SIMI development was then investigated. KEGG functional enrichment analysis was performed on 505 immune infiltration‐related SIMI genes. The results suggested that these genes were closely linked to cell adhesion molecules, complement and coagulation cascades, regulation of lipolysis in adipocytes and HIF‐1 signalling pathway (Figure 7A,B). The previous study has suggested that HIF‐1 may play an important role in SIMI development [23]. So, the HIF‐1 pathway was selected for further investigation. CCK‐8 showed that the viability of cells was considerably suppressed in the LPS + si‐BMP10 + Fenbendazole‐d3 (agonists of the HIF‐1 pathway) group in comparison with that in the LPS + si‐BMP10 group (Figure 8A). The incorporation of Fenbendazole‐d3 notably counteracted the inhibitory impact of BMP10 knockdown on apoptosis (Figure 8B). Additionally, IL‐6, MCP‐1, IFN‐β and CCL11 levels were obviously increased after the incorporation of Fenbendazole‐d3 as opposed to that in the LPS + si‐BMP10 group (Figure 8C). Results from the western blot demonstrated that the protein expressions of VCAM‐1, ICAM‐1 and HIF‐1α were substantially increased in the LPS group in comparison with that in the control group. VCAM‐1, ICAM‐1 and HIF‐1α protein expressions were notably lower in the LPS + si‐BMP10 group than in the LPS + si‐NC group, which was significantly reversed by the addition of Fenbendazole‐d3 (Figure 8D).

## 4 Discussion

Sepsis is a deadly syndrome instigated by a cascade of inappropriate immune responses. SIMI frequently occurs as a complication in individuals with severe sepsis and is correlated with heightened mortality rates [24]. In this investigation, six key genes were screened in the LPS‐stimulated SIMI model by RNA sequencing. The BMP10, HAMP, TRIM5 and MLANA expressions were considerably increased, while PTPRN2 and AVP were notably decreased. Knockdown of BMP10 inhibited apoptosis, inflammation and cell adhesion both in vivo and in vitro. Furthermore, knockdown of BMP10 suppressed the development of SIMI by inhibiting the HIF‐1α pathway, which was validated in CMECs.

LPS, found in the outer membrane of Gram‐negative bacteria, triggers the synthesis of numerous pro‐inflammatory cytokines by interacting with host effector cell receptors, potentially causing an uncontrolled inflammatory response [25]. The septic mouse model with myocardial damage induced by LPS is widely used for druggable target identification and mechanism studies [26, 27]. In this study, the SIMI mouse model was induced using LPS, and RNA sequencing was performed to identify key DEGs associated with SIMI. Consequently, there was 1521 DEGs were found between the control and SIMI mouse models, and they were mostly correlated with the response to the bacterium, immune system process, defence response and cell adhesion molecules. This suggested that SIMI was significantly correlated with the immune process. The DEGs were then taken to intersect with immune‐related genes, and six key genes were finally identified, including BMP10, HAMP, TRIM5, MLANA, PTPRN2 and AVP.

HAMP is a crucial component in maintaining iron metabolism and its elevated expression promotes ferroptosis [28]. By regulating IL‐6, conventional dendritic cells and macrophages, HAMP‐encoded hepcidin protein is involved in innate immune responses, and inflammatory and infectious processes [29, 30]. In a bioinformatics analysis, HAMP is identified as a key gene for septic cardiomyopathy and is highly expressed in LPS‐treated HL‐1 cells and the AC16 cell models [31]. TRIM5, a member of the TRIM protein family, is involved in innate immunity and acts as a barrier against specific viral infections. Its function involves recognising and breaking down the viral capsid, thereby inhibiting viral replication [32, 33]. MLANA, also referred to as melanoma antigen recognised by T cells 1, is expressed in melanocytes [34]. MLANA is a target for cancer immunotherapy, such as adoptive T cell therapy and cancer vaccines [35, 36]. PTPRN2, a member of the N‐type family of protein tyrosine phosphatase receptors, is typically found in the nervous system and endocrine cells, contributing to insulin secretion regulation [37]. PTPRN2 is associated with autoimmune diabetes [38]. AVP is a peptide hormone and its deficiency can cause polyuria‐polydipsia syndrome [39]. However, there are few investigations reported on these five key genes in SIMI. In the present investigation, we found that HAMP, TRIM5 and MLANA were highly expressed in the LPS‐induced SIMI model, and PTPRN2 and AVP were lowly expressed.

The sepsis‐induced immune response can be induced by endothelial cell dysfunction, which in turn leads to immune cell infiltration into organs and organ damage [40]. During sepsis, endothelial cells, positioned on the interior of blood vessels and in direct contact with the bloodstream, are directly exposed to bacterial metabolites or lysates, actively engaging in the inflammatory response [41]. When bacteria invade the body, macrophages secrete IL‐1, TNF‐α, IL‐6 and IL‐8 during phagocytosis of pathogens, which directly or indirectly cause myocardial injury [42]. High expression of pro‐inflammatory cytokines, such as IL‐6 and TNF‐α, promotes cardiomyocyte apoptosis and injury, which in turn promotes septic myocardial dysfunction [43]. BMP10 serves as a high‐affinity ligand for activin receptor‐like kinase 1, which is a type I BMP receptor predominantly expressed on endothelial cells [44]. Therefore, we selected BMP10 from among the six key genes for in‐depth exploration. During heart development, BMP10 is crucial for both the proliferation of cardiomyocytes and the maturation of cardiac chambers [45]. BMP10 enhances TNF‐α‐induced monocyte recruitment to vascular endothelial cells mainly through activin receptor‐like kinase 2, thereby promoting inflammation [46]. In this study, we found that knockdown of BMP10 suppressed LPS‐induced apoptosis of CMECs in the SIMI mouse model. Additionally, it led to decreased levels of inflammatory factors, including IL‐6, MCP‐1, IFN‐β and CCL11, along with decreased ECMCs immune infiltration. Furthermore, in response to inflammatory mediators, endothelial cells upregulate the expression of adhesion molecules on their surface, such as P‐selectin, E‐selectin, ICAM‐1 and VCAM‐1. This upregulation facilitates increased rolling, adherence and transmigration of leukocytes from the bloodstream into the surrounding tissue [47]. Our investigation revealed that BMP10 knockdown inhibited the protein expression of the intercellular adhesion molecules VCAM‐1 and ICAM‐1. The above findings suggest that knockdown of BMP10 inhibits the development of SIMI possibly by suppressing CMECs inflammation, apoptosis, intercellular adhesion and immune infiltration.

Subsequently, the molecular mechanism by which BMP10 influences the development of SIMI was explored. It was found that BMP10 may be significantly correlated with the HIF‐1 pathway. HIF‐1, a dimeric transcription complex, mediates the immune response. IL‐1 and TNF‐α can induce HIF‐1‐dependent gene expression even under normoxic conditions [48]. In this study, LPS treatment promoted HIF‐1α protein expression, and that knockdown of BMP10 significantly reversed this trend. Studies have shown that HIF‐1α is involved in the development of myocardial injuries. In LPS‐treated H9C2 cells, upregulation of miR‐124‐3p reduced oxidative stress, inflammation and apoptosis by regulating the SP1/HDAC4/HIF‐1α axis [49]. In sepsis‐induced myocardial dysfunction, phlorizin, a new caloric restriction mimetic, activates autophagy and protects cardiomyocytes through the HIF‐1α/BNIP3 axis [50]. In this research, we found that the inhibitory effects of BMP10 knockdown on inflammation, apoptosis and cell adhesion in the LPS‐induced SIMI model were significantly counteracted by treatment with the HIF‐1 pathway agonist Fenbendazole‐d3. This suggests that BMP10 knockdown inhibits the progression of LPS‐induced SIMI possibly by suppressing the HIF‐1α. Moreover, at present, potential immunostimulants include leukocyte growth factors, immunostimulatory cytokines, inhibitors of negative co‐stimulatory pathways such as PD1 and PDL1, and unique immunomodulators [51]. HIF‐1 has been recognised as an important cancer drug target, and inhibitors of HIF‐1 such as EZN‐2698 are potential agents for patients with advanced renal cell carcinoma [52]. In this investigation, we found that the BMP10, an immune infiltration‐associated gene, affects the development of SIMI through HIF‐1α pathway inhibition, which offers a novel potential treatment method for SIMI.

## 5 Conclusion

In this study, we found that the BMP10, HAMP, TRIM5 and MLANA expression levels were substantially elevated, and the expression levels of PTPRN2 and AVP were notably decreased. Knockdown of BMP10 inhibited inflammation, apoptosis, cell adhesion and immune infiltration of endothelial cells in LPS‐induced SIMI, which possibly through the HIF‐1α pathway inhibition. Our investigation provides novel perspectives for the immunotherapy of SIMI.

## Author Contributions

**Huan Guan:** conceptualization (equal), formal analysis (equal), methodology (equal), writing – original draft (equal). **Jingyun Fang:** formal analysis (equal), investigation (equal), writing – review and editing (equal).

## Ethics Statement

The animal experiments were approved by the local ethical committee in accordance with the ARRIVE guidelines.

## Conflicts of Interest

The authors declare no conflicts of interest.

## Supporting information


Appendix S1:

## Data Availability

The data and materials supporting the findings of this study are available from the corresponding authors upon request.
