# Supplementary material for: BMP10 Knockdown Modulates Endothelial Cell Immunoreactivity by Inhibiting the HIF‐1α Pathway in the Sepsis‐Induced Myocardial Injury
Source: J Cell Mol Med. 2024 Nov 29;28(22):e70232. doi: 10.1111/jcmm.70232 (PMC11605482; doi:10.1111/jcmm.70232)
Supplement: Supplementary file 1 — Appendix S1: [file JCMM-28-e70232-s001.docx]

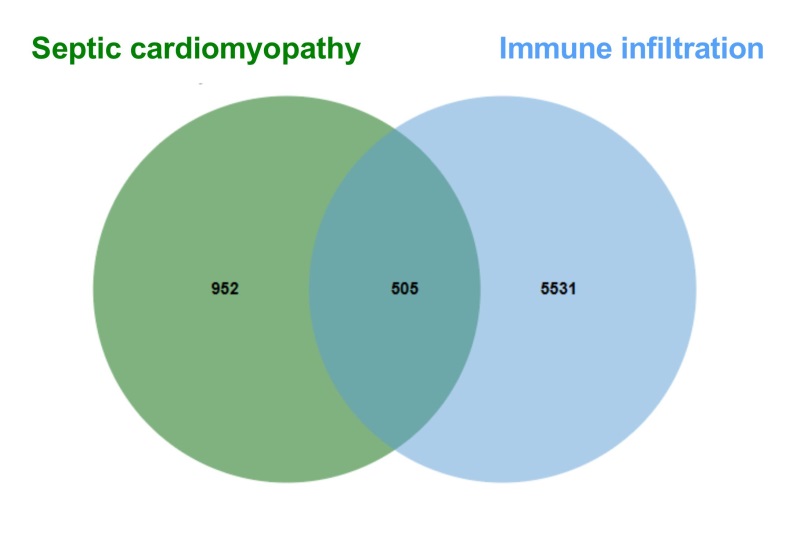


**Supplementary figure 1** Venn diagram of SIMI-associated differentially expressed genes versus immune infiltration genes. Notes: SIMI, sepsis-induced myocardial injury.

**Supplementary Table 1** Primer sequences of RT-qPCR and cell transfection used in this study

| Name | Sense | Antisense |
| --- | --- | --- |
| GAPDH | AACTTTGGCATTGTGGAAGG | ACACATTGGGGGTAGGAACA |
| BMP10 | ACTCAAAAGCCAAGCCTGAA | TTGCCAGGTAGAGGGAAATG |
| HAMP | GACCAGTGGCTCTGTTTTCC | CACATCCCACACTTTGATCG |
| TRIM5 | TCTGCACTGGTGTCTGAAGG | CAGGGGTTGTGTCAGGAGTT |
| MLANA | ATGTGAGAGCCCTGATCACC | CAGCGTTCTCAGGAGTTTCC |
| PTPRN2 | GTCCCTTCCTCCTCAAGGTC | AAACTGCTCCTTCGTCTGGA |
| AVP | CTTCCAGAACTGCCCAAGAG | GGGCAGGTAGTTCTCCTCCT |
| si-NC | UUCUCCGAACGUGUCACGUTT | ACGUGACACGUUCGGAGAATT |
| si-BMP10-1 | CGCUCUACAUCGACUUCAAGG | UUGAAGUCGAUGUAGAGCGGG |
| si-BMP10-2 | GGAUGAGUUUCUUAAGACACU | UGUCUUAAGAAACUCAUCCUU |
| si-BMP10-3 | AGUACAUGUUGGAACUCUACA | UAGAGUUCCAACAUGUACUCU |

**Supplementary Table 2** Top 10 up- and down-regulated DEGs between the SIMI mouse model and control model

| **Genes** | **Description** | **Pval.adj** | **Up/Down** | **log2FoldChange** |
| --- | --- | --- | --- | --- |
| BMP10 | bone morphogenetic protein 10 | 9.0056E-05 | Up | 14.00766878 |
| LFI213 | interferon activated gene 213 | 0.000135977 | Up | 8.182007736 |
| OLFR1033 | olfactory receptor 1033 | 1.52022E-29 | Up | 7.921454358 |
| HAMP | hepcidin antimicrobial peptide | 0.009416099 | Up | 7.588297088 |
| TRIM5 | tripartite motif-containing 5 | 4.83983E-13 | Up | 7.480727001 |
| RPL29 | ribosomal protein L29 | 2.3306E-31 | Up | 7.29302703 |
| MLANA | melan-A | 0.04678425 | Up | 6.93470844 |
| ISOC2B | isochorismatase domain containing 2b | 6.38149E-20 | Up | 6.683800366 |
| SAA3 | serum amyloid A 3 | 0.010354461 | Up | 6.644366118 |
| RPL26 | ribosomal protein L26 | 6.35982E-42 | Up | 6.569546241 |
| AQP6 | aquaporin 6 | 2.3306E-31 | Down | -11.63383796 |
| GBP2B | guanylate binding protein 2b | 6.6919E-27 | Down | -8.0165092 |
| MINDY4B-PS | MINDY lysine 48 deubiquitinase 4B, pseudogene | 1.83162E-55 | Down | -7.99584921 |
| AK157302 | cDNA sequence AK157302 | 1.57939E-71 | Down | -6.438761576 |
| PTPRN2 | protein tyrosine phosphatase, receptor type, N polypeptide 2 | 6.35982E-42 | Down | -6.000148285 |
| H2BC24 | H2B clustered histone 24 | 0.004076181 | Down | -5.858058589 |
| AVP | arginine vasopressin | 0.005542454 | Down | -5.802513788 |
| SLC28A2B | solute carrier family 28 member 2b | 2.5967E-19 | Down | -5.545943274 |
| IFI202B | interferon activated gene 202B | 0.000769543 | Down | -5.51668483 |
| ACSM2 | acyl-CoA synthetase medium-chain family member 2 | 0.000276423 | Down | -5.403866791 |
